# The utility of different screening methods to detect hazardous drinking and alcohol use disorders in the Screening and Intervention Program for Sensible Drinking (SIPS) program

**DOI:** 10.1186/1940-0640-7-S1-A83

**Published:** 2012-10-09

**Authors:** Simon Coulton, Colin Drummond, Paolo Deluca, Eileen Kaner, Dorothy Newbury-Birch, Katherine Perryman, Tom Phillips

**Affiliations:** 1 Center for Health Service Studies, University of Kent, Canterbury, UK; 2 National Addiction Center, Institute of Psychiatry, King's College London, London, UK; 3 Institute of Psychiatry, King's College London, London, UK; 4 Institute of Health and Society, Newcastle University, Newcastle upon Tyne, UK; 5 School of Medicine, University of Manchester, Manchester, UK; 6 Humber National Health Service Foundation Trust, Willerby, UK

## 

Numerous screening methods have been developed to detect hazardous and harmful drinking in a range of health settings. Recent research has focused on developing briefer screening tools to maximize implementation in busy practice settings, particularly emergency departments (EDs) and primary care. However the relative utility of these tools is not fully understood. Further, there is a need to identify the utility of universal screening, in which all patients approaching primary care are screened, compared with targeted screening, which includes only patients with certain “red flag” conditions or presentations. The Screening and Intervention Program for Sensible Drinking (SIPS) program compared the relative utility of different screening tools (e.g., the Single Alcohol Screening Question [SASQ] and the Fast Alcohol Screening Test [FAST]) and approaches (universal versus targeted screening) in primary care. In addition, the utility of the Paddington Alcohol Test (PAT), a targeted screening tool, was compared with SASQ and FAST in EDs. Compared with the Alcohol Use Disorders Identification Test (AUDIT), the FAST had a higher sensitivity than the SASQ in primary care. Although targeted screening in primary care is a more efficient screening method, it misses a large proportion of patients who could benefit from brief interventions. The SASQ performed better in EDs than either the FAST or PAT. These results have important implications for the choice of screening tools in different settings.

